# Effects of use of an eHealth platform e-Vita for COPD patients on disease specific quality of life domains

**DOI:** 10.1186/s12931-019-1110-2

**Published:** 2019-07-10

**Authors:** Esther P.W.A. Talboom-Kamp, Marije S. Holstege, Niels H. Chavannes, Marise J. Kasteleyn

**Affiliations:** 1 Public Health and Primary Care Department, Leiden University Medical Center, Leiden, Netherlands; 2 National eHealth Living Lab, University of Leiden, Leiden, Netherlands; 3 Saltro Diagnostic Center, Utrecht, Netherlands; 4 Department of Research, Treatment and Advice Center Omring GRZPLUS, Hoorn, The Netherlands; 5 Department of Research and Development, Evean, Purmerend, The Netherlands

## Abstract

**Background:**

Integrated disease management with self-management for Chronic Obstructive Pulmonary Disease (COPD) is effective to improve clinical outcomes. eHealth can improve patients’ involvement to be able to accept and maintain a healthier lifestyle. Eventhough there is mixed evidence of the impact of eHealth on quality of life (QoL) in different settings.

**Aim:**

The primary aim of the e-Vita-COPD-study was to investigate the effect of use of eHealth patient platforms on disease specific QoL of COPD patients.

**Methods:**

We evaluated the impact of an eHealth platform on disease specific QoL measured with the clinical COPD questionnaire (CCQ), including subscales of symptoms, functional state and mental state. Interrupted time series (ITS) design was used to collect CCQ data at multiple time points. Multilevel linear regression modelling was used to compare trends in CCQ before and after the intervention.

**Results:**

Of 742 invited COPD patients, 244 signed informed consent. For the analyses, we only included patients who actually used the eHealth platform (*n* = 123). The decrease of CCQ-symptoms was 0.20% before the intervention and 0.27% after the intervention; this difference in slopes was statistically significant (*P* = 0.027). The decrease of CCQ-mental was 0.97% before the intervention and after the intervention there was an increase of 0.017%; this difference was statistically significant (*P* = 0.01). No significant difference was found in the slopes of CCQ (*P* = 0.12) and CCQ-function (*P* = 0.11) before and after the intervention.

**Conclusion:**

The e-Vita eHealth platform had a potential beneficial impact on the CCQ-symptoms of COPD patients, but not on functional state. The CCQ-mental state remained stable after the intervention, but this was a deterioration compared to the improving situation before the start of the eHealth platform. Therefore, health care providers should be aware that, although symptoms improve, there might be a slight increase in anxiety and depression after introducing an eHealth intervention to support self-management.

**Trial registration:**

Our study is registered in the Dutch Trial Register (national registration of clinical trails, mandatory for publication) with number NTR4098 and can be found at http://www.trialregister.nl/trial/3936.

Date registered: 2013-07-31.

First participant: 2014-01-01.

**Electronic supplementary material:**

The online version of this article (10.1186/s12931-019-1110-2) contains supplementary material, which is available to authorized users.

## Background

The number of individuals with a chronic illness is growing rapidly due to the ageing population and longer individual life span. Chronic illnesses are expected to be the primary cause of death and disability in the world by 2020 [1]. This increase leads to a substantial impact on society, a high burden on patients’ lives and a higher workload in health care [2–6]. Therefore structural changes of the organisation of the healthcare system are needed, with an important role for developing self-management for patients using eHealth [7]. Studies have shown that eHealth interventions are effective in stimulating self-management and reducing medical staff consultations [8, 9].

Chronic obstructive pulmonary disease (COPD) is a slowly progressive lung disease ranging from mild to very severe based on the severity of symptoms. COPD represents one of the main causes of morbidity and mortality worldwide [10]. COPD was responsible for 6% of all deaths in 2012 [11, 12]. Patients show a large variation in clinical presentations, and limitations in daily life, therefore the management of COPD is highly complex. Major treatment goals in COPD are prevention of disease progression, reduction of mortality, reduction of symptoms, improvements of exercise tolerance and health status, and prevention and treatment of complications and exacerbations [13]. Treatment is also focused on optimising the quality of life (QoL) and daily functioning of patients.

Self-management support of COPD as part of integrated disease management is an effective method to improve the quality and efficacy of care and to reduce healthcare costs [2, 14, 15]. Self-management support can improve QoL, exercise capacity and reduce hospital admissions and number of sick days because of exacerbations [16]. Self-management among patients in pulmonary rehabilitation with moderate to severe COPD has the potential to have a higher impact on cost saving due to reducing use of health care services [17], results in primary care remain inconclusive [18]. EHealth is considered to have a great potential in support self-management. However, large scale implementation of eHealth for self-management support still lags behind, despite the growing need for structural changes in primary and secondary health care delivery [17].

Because of the importance of developing self-management using eHealth, we designed a multilevel study “e-Vita COPD” to investigate the impact of a self-management web platform to support patients with COPD in primary and secondary care [7]. The web platform provides continuous education and contact with healthcare professionals aiming to stabilise their QoL by improving self-management of exacerbations in an early phase. Previously, we showed that this program had no beneficial impact on QoL after 15 months in primary care [19]. One of our explanations was that it takes more time than 15 months to change behaviour, so QoL improvements are more likely after a longer follow-up period. However, it can be thought that certain domains of QoL change do not imply behavioural changes, and therefore can change more rapidly. Hence, an effect on these domains can be expected within 1 year.

Therefore, the main aim of the secondary analyses of this study was to analyse the effects of use of a patient platform on disease specific quality of life, symptoms, functional state and mental state after implementation of a platform for COPD patients. Furthermore, we evaluated the usage of the platforms in the different groups.

## Methods

### Study design and setting

The primary aim of the e-Vita-COPD-study was to investigate the effect of use of patient platforms on clinical outcomes of COPD patients. The e-Vita-study included 3 different care groups in primary care (groups 1, 2 and 3) and 1 pulmonary rehabilitation group (group 4); all patients started using web-based platforms on top of their usual care [7]. The study was an implementation study with a prospective parallel cohort design. We chose for an interrupted time series (ITS) design to evaluate the disease specific quality of life (QoL) within each group. The measurement of disease specific QoL started with 3 measurements at a 2 weeks frequency 1 month before the intervention, and 9 measurements until 15 months after the intervention.

In groups 1, 2 and 4, we offered the patients blended care, and in group 3 the self-management platform was offered to the patients as an independent module. Differences between the groups [1–3] are previously described in detail [7, 19]. In summary, in group 1, the online platform was offered as a highly integrated part of the COPD integrated disease management with a tailored intensive training on COPD and eHealth for healthcare professionals. Group 2 had a medium level of integration with a basic training on COPD and eHealth for health care professionals. In pulmonary rehabilitation group 4 all respiratory nurses were trained to use the platform and to communicate with patients according to the principles of self-management. The trainings were developed and provided by the e-Vita study group and are based on national and international guidelines. The respiratory nurses also played an important role in development of the platform, such as the integration of bronchodilator protocols for patients. In group 1, 2 and 4 different levels of assistance -home visits or telephone consultations by a research nurse- were offered, while group 3 received no assistance at all.

### Participants

Three primary care groups participated in the e-Vita-study; COPD patients of general practices in these care groups were eligible. In group 4 a pulmonary rehabilitation group participated in the e-Vita study; COPD-patients in this group were referred by a pulmonologist after hospital discharge.

More specifically, for all groups, patients were eligible when they were diagnosed with COPD according to GOLD criteria (post-bronchodilator FEV1/FVC < 0.7) in accordance with the Dutch general practitioners (GPs) COPD [20] and when they were treated for COPD in primary care. The study was intended to be inclusive rather than exclusive to achieve high external validity (applicability to daily practice). Patients were excluded if they were unable to fill in questionnaires, patients that had no access to internet, patients with terminal illness, immobile patients and patients with severe substance abuse.

### Recruitment

We recruited primary care groups by inviting GPs in groups 1, 2 and 3 to participate. For group 4, we recruited a pulmonary rehabilitation group to participate in our study by inviting the referring pulmonologists.

Patients were invited to participate by letter via their own GP or their pulmonologist. When participants of the e-Vita study logged in and used the Web platform at least once, they were defined as users. Patients were defined as lost to follow-up if they did not log on to the platform for at least 12 months after signing informed consent, or if they did not complete the digital questionnaires within the intervention period.

### Intervention

The intervention consisted of a combination of a web-based COPD-specific platform for patients, different trainings for healthcare professionals, and several levels of guidance by a nurse for the patients.

The web-based platform provided disease specific education and tips that fit their personal disease management program. In addition, the platform provided tools to report and monitor personal health goals, actions and health-related QoL that could be shared with the patients’ own practice or respiratory nurses (Additional file 1). The patient platform could be used by healthcare professionals to prepare consultation or to monitor patients in-between their visits to their general practice.

In the primary care groups 1 and 2 all healthcare professionals were trained; the training program in group 1 and 4 was very thorough and in group 2 basic knowledge was provided. In group 3 healthcare professionals did not receive any training. In group 1, 2 and 4 COPD-patients were invited for a personal intake and explanation about the platform. In group 3 patients received a written instruction.

Participants all received usual COPD care beside using the platform.

### Outcome measures

The outcomes of usage included number of sessions and services. A session was defined as a period between logging in and logging out of the e-Vita platform and a service was defined as a focused action within the platform [7, 19].

The primary outcomes were disease specific QoL domains, measured with the subscales of the clinical COPD questionnaire (CCQ) [21]. CCQ was measured with a 2 weeks interval conform the ITS design, 3 times before the intervention (− 4, − 2 and 0 weeks) and 9 times after the intervention; 3 times at 26 weeks, 3 times at 52 weeks and 3 times 65 weeks. The CCQ is an instrument to measure disease specific QoL in patients with COPD and consists of 10 items, each scored on a 7-point Likert scale. The total scores are calculated as the mean of the sum of all items and ranges from 0 to 6, with higher scores representing a lower QoL. The CCQ comprises three domains: symptom state (4 items), functional state (4 items) and mental state (2 items). Total scores for each of these domains also range from 0 to 6.

### Statistical methods

For the exploratory post hoc analyses described in this manuscript, we performed no formal sample size calculation. Normally distributed continuous variables are reported as means with standard deviations (SD), non-normally distributed continuous variables as medians with interquartile ranges (IQR) and categorical variables as number and percentages. All patients who logged in at least once on the platform were included in the analyses.

Multiple linear regression analyses were performed to evaluate the differences in use of the e-Vita COPD platforms between the four groups. For every month during the intervention period, attrition was measured by logging and evaluating the percentage of users that used the platform. The area under the curve was calculated for a period of 18 months; after this period, usage dropped to zero for two groups.

To analyse the effects of the usage of the platforms on the CCQ and the subdomains of the CCQ, time trends before and after intervention were studied using ITS analyses. Since we had repeated measurements within a patient, we used multilevel linear regression modelling (mixed models). The analyses allowed us to quantify the effect of the intervention on CCQ (subdomains) versus the observed pre-intervention period. Estimates for regression coefficients corresponding to 2 standardised effect sizes were obtained: a direct change in the level of the CCQ (also called step change or jump) and a change in trend of the CCQ before and after the intervention [22]. Included in the four models (total CCQ score, symptom state, functional state and mental state) the fixed effects were time, treatment, and the interaction between time and treatment; All models included a random intercept per patient. When there was a substantial improvement in the Akaike Information Criterion (used to assess the model fit score), an additional random slope (time) was used.

All analyses were performed with SPSS version 23.0 (IBM Corporation, Armonk, NY, USA).

## Results

Of the 702 COPD patients invited via their primary care groups, 215 signed informed consent. Of the 40 COPD patients invited via their pulmonologist, 29 signed informed consent. For the analyses, we included the patients who actually used the eHealth platform (*n* = 123). Baseline characteristics of the included COPD population are listed in Table 1. The pulmonary rehabilitation group (group 4: median 2.9 IQR [2.3–3.7]) showed higher baseline CCQ scores, indicating a more severe health status in comparison with the primary care groups (group 1–3) (group 1: median 1.2 IQR [0.7–1.8]; group 2: median 1.6 IQR [0.97–2.23]; group 3: median 0.9 IQR [0.4–1.6]).

**Table 1:** Baseline characteristics of the users

	Primary care group 1	Primary care group 2	Primary Care group 3	Pulmonary rehabilitation group 4	Total
	*n* = 43	*N* = 42	*n* = 21	*n* = 17	*n* = 123
Age in years, median [IQR]	64.1 [60.1–73.4]	66.9 [59.9–74.9]	64.4 [62.1–69.9]	69.3 [62.4–78.0]	66.3 [60.3–73.4]
Male gender, n (%)	27 (62.8)	16 (38.1)	11 (52.4)	11 (64.7)	65 (52.8)
Baseline CCQ, median [IQR]	1.2 [0.7–1.8]	1.6 [0.97–2.2]	0.9 [0.4–1.6]	2.9 [2.3–3.7]	1.3 [0.7–2.3]

*IQR* Interquartile rang, *CCQ* Clinical COPD Questionnaire

### Usage of the web-based platforms

**Fig. 1:**
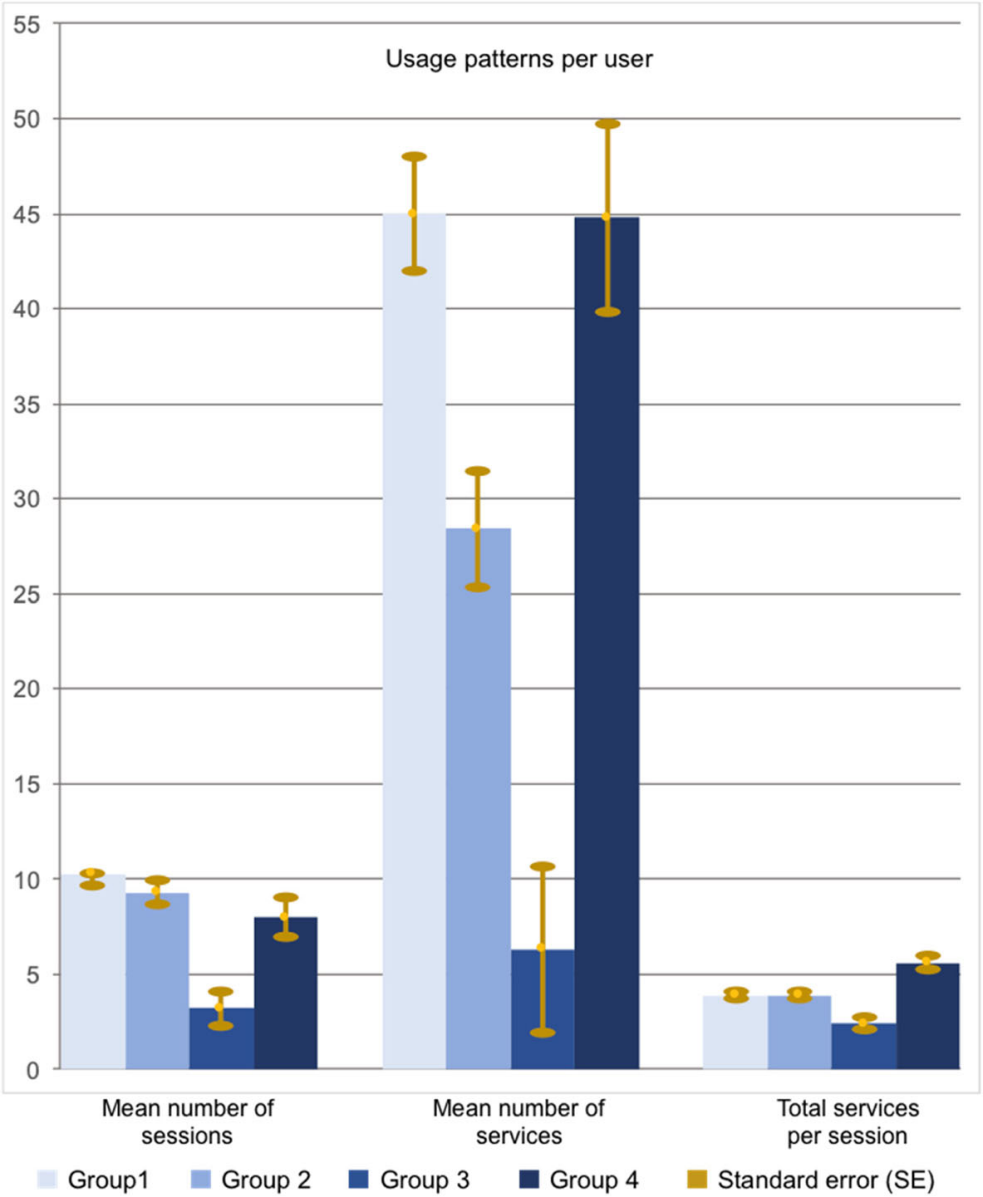
Usage patterns in each group

Figure 1 shows the use of the COPD-platforms for all groups. The mean number of sessions per user differed between the four groups (group 1: mean 10.3, SE 1.3; group 2: mean 9.3, SE 1.3; group 3: mean 3.2, SE 1.8; group 4: mean 8.0, SD 2.1; *P* = 0.016 *F* = 3.6). Also the mean number of services per user differed between the groups (group 1: mean 45.0, SE 6.1; group 2: mean 28.4, SE 6.2; group 3: mean 6.3, SE 8.7; group 4: mean 44.8, SE 9.9; *P* = 0.002 *F* = 5.1) and the number of services per session per user was also different between the four groups (group 1: mean 3.9, SE 0.4; group 2: mean 4.1, SE 0.4; group 3: mean 2.1, SD 0.6; group 4: mean 5.6, SE 0.7; *P* = 0.008 *F* = 4.1).

### Changes in quality of life

**Fig. 2:**
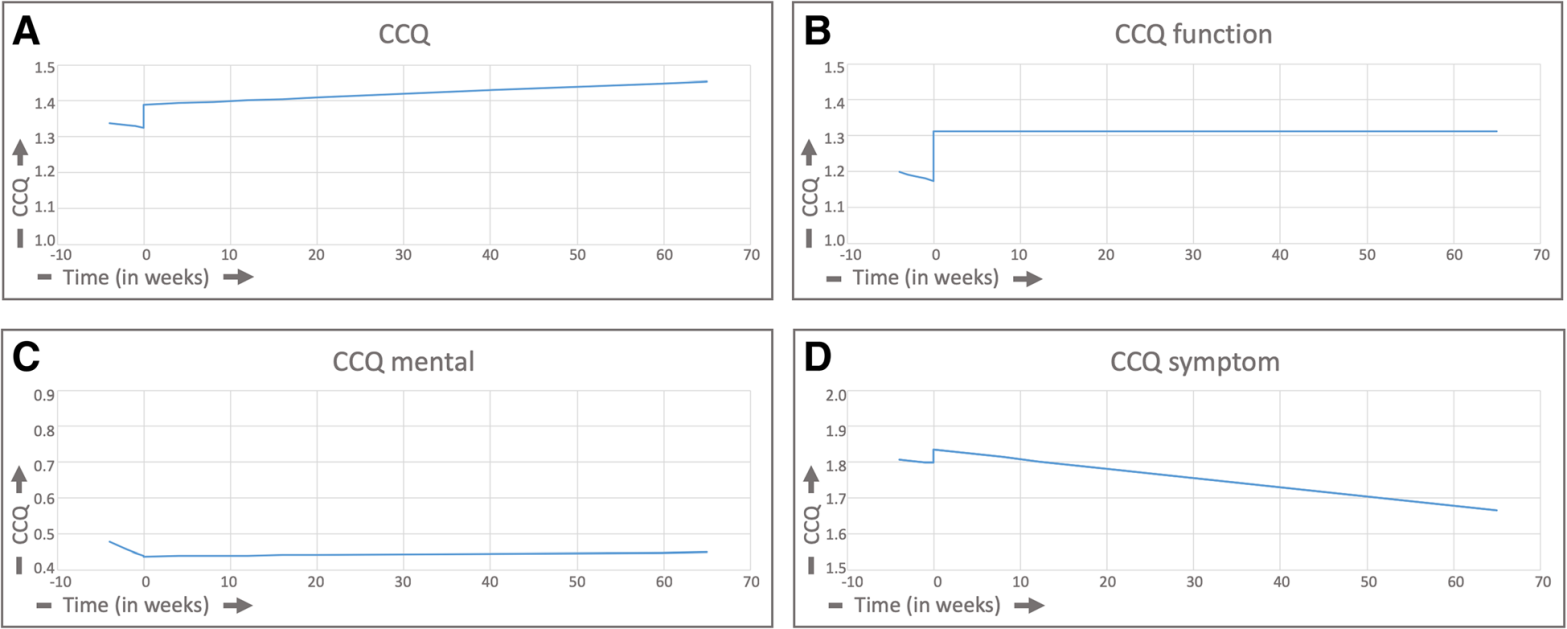
**a** Total group analysis of CCQ, **b** total group analysis of CCQ-functional state, **c** total group analysis of CCQ-mental state, **d** total group analysis of CCQ-symptoms

Figure 2a shows the effect of the intervention on total CCQ score. The decrease in CCQ before the intervention was 0.3% per month and the increase after the intervention 0.1% per month; this difference in trends was not significant (*P* = 0.31). The estimated direct change in the level of the CCQ slopes between before and after the intervention at the moment of the start of the intervention (jump) was 0.027 (*P* = 0.12) implying that the CCQ trend was 2.7% lower before the intervention.

Figure 2b shows the effect of the intervention on CCQ-function. The decrease before the intervention was 0.6% per month and the increase after the intervention 0.001% per month; this difference was not significant (*P* = 0.24). The estimated direct change in the level of the CCQ function slopes at the moment of the intervention (jump) was − 0.06 (*P* = 0.011) implying that the CCQ function trend was 6% lower before the intervention.

Figure 2c shows the effect of the intervention on CCQ-mental state. The decrease before the intervention was 0.97% per month and the increase after the intervention 0.01% per month; this difference in slopes was significant (*P* = 0.014). The estimated direct change in the level of the CCQ-mental slopes at the moment of the intervention (jump) was 0.0005 (*P* = 0.984) implying that the CCQ-mental trend was 0.05% higher before the intervention.

Figure 2d shows the effect of the intervention on CCQ-symptom. The decrease of CCQ-symptoms was 0.2% before the intervention and 0.3% after the intervention; this difference in slopes was statistically significant (*P* = 0.027). The estimated direct change in the level of the CCQ-symptom slopes at the moment of the intervention (jump) was − 0.013 (*P* = 0.51) implying that the CCQ-symptom trend was 1.3% lower before the intervention.

### Attrition

**Fig. 3:**
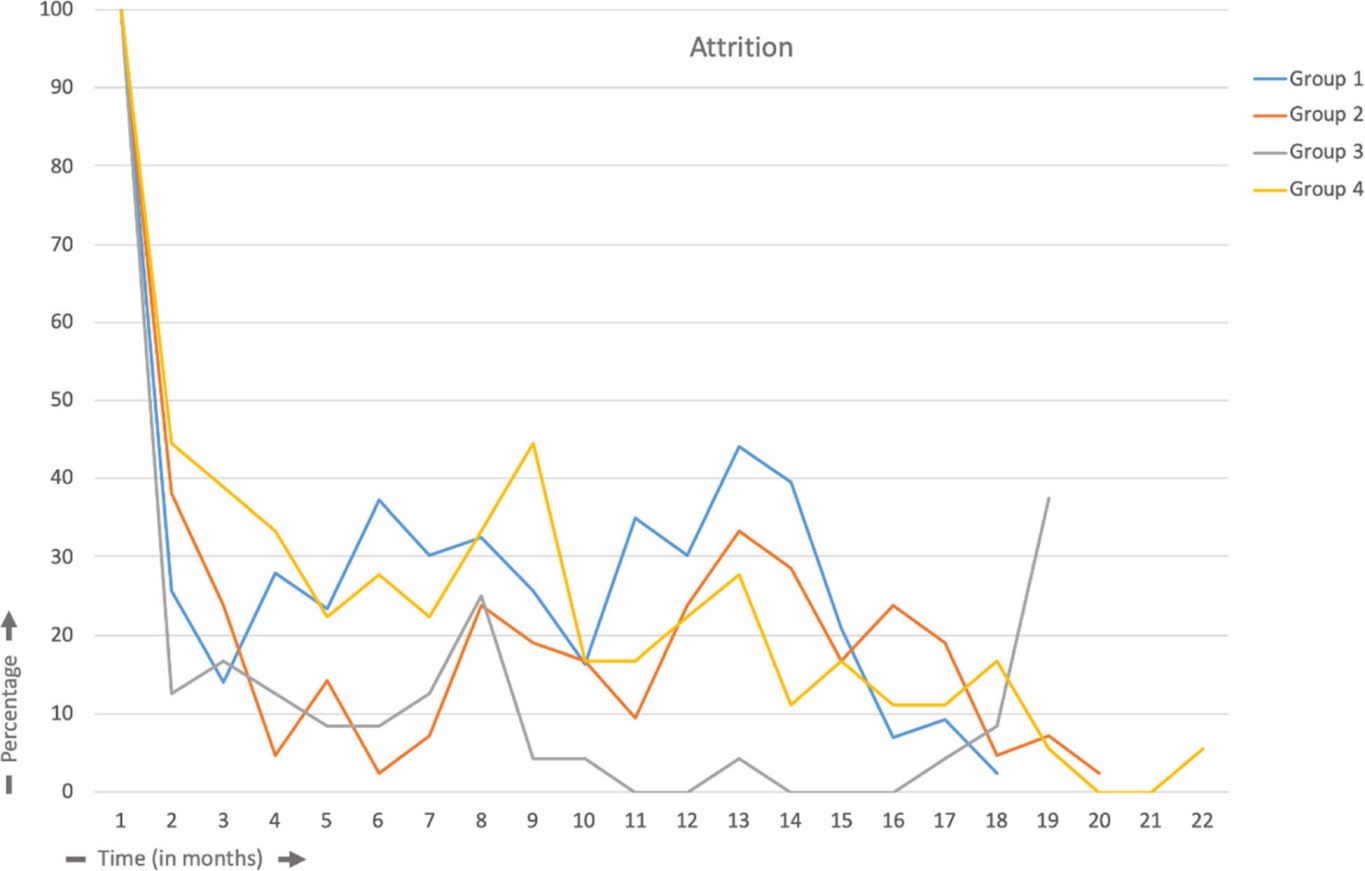
Attrition

The log files revealed that a substantial proportion of the users did not continuously use the platforms before completion of the study. Figure 3 shows the patterns of use of the eHealth platforms in groups 1 to 4 during the intervention period, with the percentage of users on the y-axis, starting with 100% of the users, and the duration of usage in months on the x-axis. The area under the curve until the 18th month for attrition in group 1–4 was 469.9; 357.1; 124.1; 458.3 respectively.

## Discussion

### Main findings

The use of eHealth solutions is not yet common practice for chronic patients, although expectations remain high and a growing number of healthcare professionals is experimenting with eHealth. Our primary aim was to analyse the impact of usage of an eHealth platform on domains disease specific QoL. We found that usage of the COPD self-management Web-based platform was higher when the platform was an integrated part of usual care with trained health care professionals who encourage patients to use the platform. No changes in total CCQ were found after introduction of the eHealth-supported COPD programs. The subscales of CCQ which reflect the mental state and the symptoms differed significantly before and after introduction of the program; the CCQ-mental slightly increased after the intervention (decreasing trend before the intervention) and the CCQ-symptom decreased significantly more after the intervention than before the intervention.

Our findings highlight the importance of integrating eHealth into usual care; usage of the platform is higher when the platform is an integrated part of a care program with appropriate personal coaching for patients. Similar results were found in an earlier study on COPD and asthma patients; the online app was used on a more regular basis with higher involvement of the health care provider and more assistance of the patients [23]. The e-Vita study on patients with diabetes mellitus showed minimal impact of implementing a personal health record including self-management support in primary diabetes care; recommendations were made to use additional strategies for patient motivation and engagement of professionals for a successful adoption of Web-based platforms [24, 25]. In the current study, we organised extensive professional training of health care professionals on COPD and self-management supported by eHealth; we also offered personal assistance for the users to guide them through the platform. Both strategies are essential elements to influence the use of platforms.

The changes in total scores of CCQ after 15 months were not within the range of a minimal clinically important difference (MCID) of 0.4 points [26]. CCQ is determined by a significant number of factors [27]. We expect that eHealth interventions will be effective in stabilising and improving QoL in COPD patients when these patients use the platforms for a longer period of time.

Since self-management skills imply behavioural change which requires a longer time, we expected to be able to detect changes in disease specific QoL domains. The significant flattening of the improvement of CCQ-mental state after introducing the intervention might be explained by the participant rise in consciousness regarding their disease; symptoms of anxiety and depression are common among COPD patients, and the right treatment remains inconclusive [28]. On the other hand, the domain symptoms improved after introducing the intervention. The significant decline in the decrease of CCQ-symptoms after the intervention, which means an improvement of the COPD-symptoms, is described in other studies with improving symptoms after the introduction of self-management [29, 30]. COPD-patients have reported that the relief of symptoms like shortness of breath, is most important for them when starting with treatment; the improvement of CCQ-symptoms in our study meets the wishes of COPD-patients [31]. Because of our ITS-design with a mixed-models statistical analysis, we detected a changing trend of the CCQ-subscales mental state and symptoms. However, it does not necessarly mean that these findings are also clinically relevant. Unfortunatly, for the subscales of the CCQ no MCIDs are reported. Also, extrapolation of slopes towards MCIDs have not been described in literature. Eventhough, we expect that the symtoms will be clinically better after a longer period of time with eHealth, but further studies are needed to confirm this. Regarding functional state, we found no differences in trends before and after the intervention. In other studies self-management interventions also had no impact on functional state [30, 31]. It can be thought that changes in functional state are proceeded by behavioural changes and improvements regarding mental and symptom state, and that it therefore requires more time to find an impact on functional state. Therefore, health care providers should be aware that although the implementation of an eHealth platform has a beneficial effect on symptoms, there might be a slight increase in anxiety and depression. Also, since an effect on function seems unlikely, we advise health care providers and patients to take this into account when considering action plans or personal goals.

The immediate change in level of the total score of CCQ and the symptomatical and functional subscales (positive) at the start of the intervention might be explained by the participant rise in consciousness regarding their health status, thereby completing the questionnaire more critically after explanation from a health care professional. Similar to our study, in a randomized controlled trial (RCT) with asthma patients, the QoL changed immediately after starting to use a self-management portal [32].

Analysis of attrition provided insight into the decrease in usage (e.g., after 1 month, 10–45% of the participants were actively using the platform). The attrition was measured amongst the patients that actually started with the intervention. The periodic steep rise in the percentage of users might be explained by the email reminders sent by the platform to fill in the questionnaires; all users received continuous reminders during the intervention period. In group 3, all users received urgent and repeated requests to fill in questionnaires at the end of the intervention period, which probably explains the steep rise in the percentage of users at the end of the study. The attrition curve depicts the “push factors” that are required to remind participants to use the platform. This “law of attrition” (the phenomenon of participants stopping usage) is a common finding in eHealth evaluations and one of the fundamental and methodological challenges in the evaluation of eHealth apps [33].

In literature, several studies focused on predictors of eHealth usage. In a process analysis of the actual usage of web-based applications, it became clear that innovations in health care will diffuse more rapidly when technology is employed that is simple to use and has applicable components for interactivity [34]. For clinically significant improvements in diabetes self-management a range of components need to be incorporated into telehealth interventions: patient education, health care provider education, self-monitoring profile, blood test goals, easy use of blood diagnostic data to modify behavior, feedback to patients, and 2-way interaction [35]. These components are relevant for all chronic illnesses and should be incorporated in platforms. In the version of the e-Vita platform used in our study, these components were insufficiently incorporated.

In more recent studies an agile development and evaluation approach which is multidisciplinary, is recommended [36]. It is recommended to start with user experience design, development and (beta) testing, followed by clinical trial evaluation and post-market surveillance [36]. Our research team had an active role to involve patients in several development phases of the e-Vita platform; patients commented on the usability of the platform. Patients were especially dissatisfied with the log-in procedure; they also commented on the disease-centered approach. The platform was adapted after these sessions. Our study also contributed to the clinical evaluation of the platform. After our experience in eHealth research we strongly recommend a patient-centered approach with an important role for patients in all phases of the development and evaluation of eHealth. Input of patients is indispensable for sustainable eHealth solutions.

### Strengths and limitations

To the best of our knowledge, this study is the first to evaluate the effect of the use of an eHealth platform on the subscales of the CCQ. This gives valuable information regarding in which domains improvements, or deteriorations, can be expected on the short time after introducting an eHealth intervention. Another strength of this study was that participants were recruited from four different settings with different baseline scores on the CCQ. Therefore, results of this study are generalisable to different populations and settings. Furthermore, an ITS analysis was used, which is able to detect changes in trend lines before and after the intervention.

There were also some limitations. First, for the post-hoc exploratory analyses used in this study no formal sample size calculations were performed and especially in the third and fourth group the number of participants were small. However, the main analyses were performed in the total group, with a much larger sample size. Second, patients could decide whether to participate in this study or not. This probably resulted in relatively motivated participants and therefore, results are mainly generalisable to motivated persons. Also, as in most eHealth studies, there was attrition, especially in the group participating without support from their health care provider. Third is that we have no information regarding differences in baseline therapy between the three primary care groups. Therefore, it cannot be excluded that baseline differences contribute to the finding that usage differs per group. Nevertheless, since all GPs are providing usual care according to the guidelines, we expect that baseline differences are minimal.

## Conclusion

The e-Vita eHealth platform had a beneficial impact on the CCQ-symptoms of COPD patients, but not on functional state. The CCQ-mental state remained stable after the intervention, but this was a deterioration compared to the improving situation before the start of the eHealth platform. Health care providers should be aware that, although symptoms improve, there might be a slight increase in anxiety and depression after introducing an eHealth intervention to support self-management.

## Additional file

Additional file 1: Homepage e-Vita. (JPG 179 kb)

## Data Availability

The datasets used and/or analysed during the current study are available from the corresponding author on reasonable request.

## Abbreviations

CCQ: Clinical COPD Questionnaire
COPD: Chronic Obstructive Pulmonary Disease
GPs: General Practitioners
ITS: Interrupted Time Series
QoL: Quality of Life
