# Supplementary figures and images for: Effects of use of an eHealth platform e-Vita for COPD patients on disease specific quality of life domains
Source: Respir Res. 2019 Jul 10;20:146. doi: 10.1186/s12931-019-1110-2 (PMC6621945; doi:10.1186/s12931-019-1110-2)

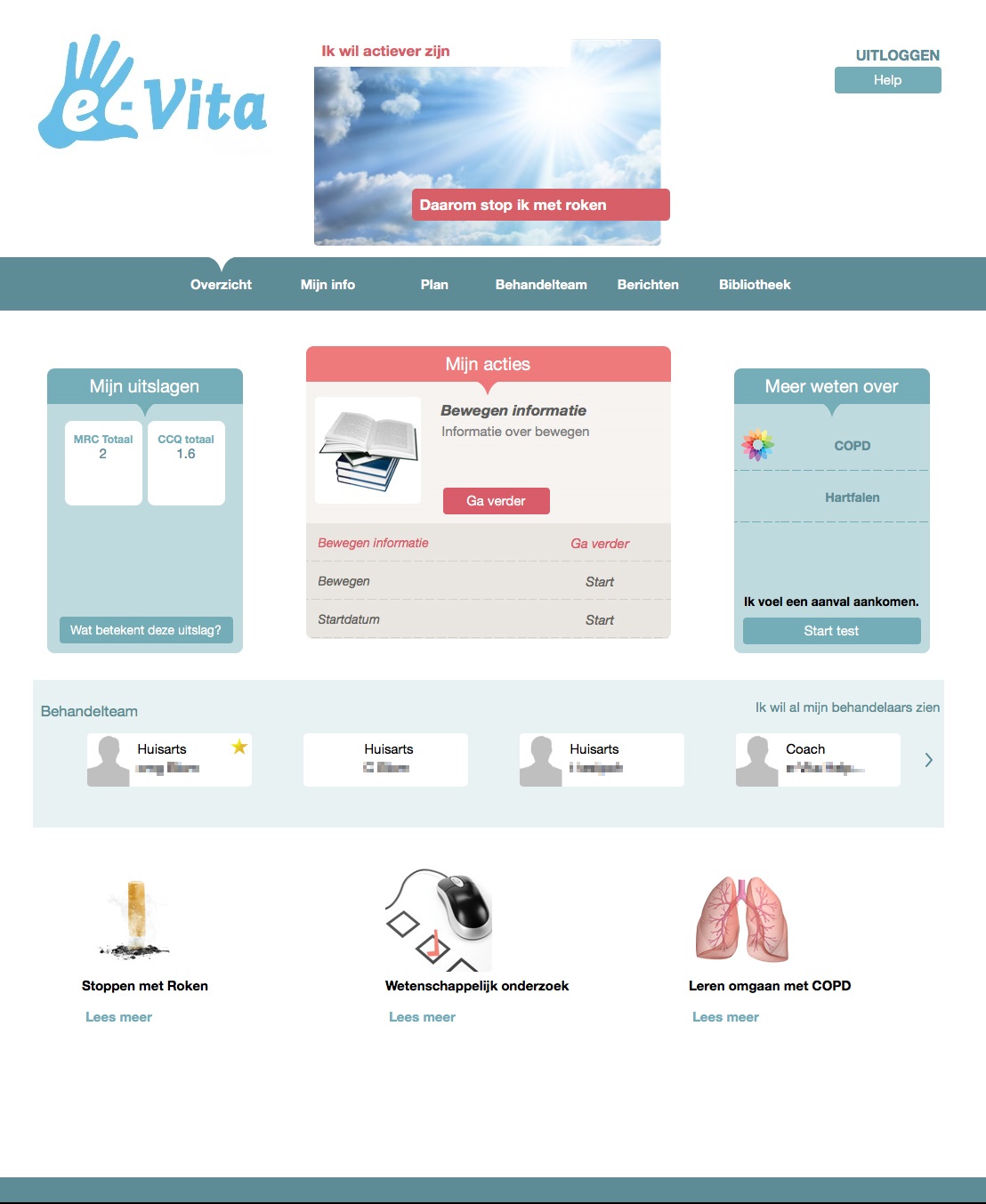

Supplement: Supplementary file 1 — Homepage e-Vita. (JPG 179 kb) [file 12931_2019_1110_MOESM1_ESM.jpg]
